# Associations of taste sensitivity with frailty and health-related quality of life in older adults

**DOI:** 10.1016/j.jnha.2026.100794

**Published:** 2026-01-24

**Authors:** Sarasa Kato, Megu Y. Baden, Eri Yamabayashi, Saki Kawamoto, Takuya Kagisaki, Kento Mitsushio, Akiko N. Beppu, Naoko Nagai, Tomomi Horii, Chisaki Ishibashi, Yoshiya Hosokawa, Mitsuyoshi Takahara, Junji Kozawa, Hitoshi Nishizawa, Toshihiro Takeda, Shunsuke Yamaga, Mashu Kudoh, Daiki Kurita, Masae Kuboniwa, Iichiro Shimomura

**Affiliations:** a Department of Metabolic Medicine, Graduate School of Medicine, The University of Osaka, Suita, Osaka, Japan; b Department of Lifestyle Medicine, Graduate School of Medicine, The University of Osaka, Suita, Osaka, Japan; c Department of Diabetes Care Medicine, Graduate School of Medicine, The University of Osaka, Suita, Osaka, Japan; d Department of Metabolism and Atherosclerosis Medicine, Graduate School of Medicine, The University of Osaka, Suita, Osaka, Japan; e Department of Medical Informatics, Graduate School of Medicine, The University of Osaka, Suita, Osaka, Japan; f Department of Preventive Dentistry, The University of Osaka Dental Hospital, Suita, Osaka, Japan; g Department of Preventive Dentistry, Graduate School of Dentistry, The University of Osaka, Suita, Osaka, Japan

**Keywords:** Taste sensitivity, Frailty, Health-related quality of life, Diet quality, Oral function

## Abstract

**Background:**

Reduced taste sensitivity may contribute to appetite loss and frailty in older adults, yet evidence based on objectively measured taste sensitivity is limited.

**Objective:**

To examine the associations of objectively measured taste sensitivity with frailty, health-related quality of life (HR-QoL), diet quality, and oral function.

**Methods:**

We assessed sweet, salt, and umami taste sensitivities in 70 community-dwelling older adults (mean age 82 years) using the whole-mouth method. Frailty was evaluated using the Basic Checklist, HR-QoL was assessed using the SF-36, diet quality was assessed using the Modified Japanese Diet Score (MJDS), and oral function was assessed by dentists using established criteria. Multiple regression analyses were performed to assess the associations of taste sensitivity with frailty, HR-QoL, diet quality, and oral function.

**Results:**

Lower sweet taste sensitivity was associated with greater frailty (coefficient [95% CI], 0.28 [0.10, 0.46], p = 0.004). Lower sweet and umami taste sensitivities were significantly associated with lower mental HR-QoL (−6.47 [−11.44, −1.50], p = 0.01 and −5.89 [−11.40, −0.38], p = 0.04, respectively). Higher diet quality and better oral function tended to be associated with better sweet taste sensitivity.

**Conclusions:**

Sweet taste sensitivity is inversely associated with frailty and mental HR-QoL. Improving diet quality and oral function may contribute to improved taste sensitivity.

## Highlights

• Low sweet taste sensitivity was associated with frailty.
• Low sweet and umami sensitivities were related to lower mental HR-QoL.
• A healthy Japanese diet tended to be associated with better sweet taste sensitivity.
• Better oral function tended to be associated with higher sweet taste sensitivity.
• Improving diet quality and oral function may help maintain taste and prevent frailty.

## 1 Introduction

Healthy life expectancy is defined by the World Health Organization as a period of time during which one's daily life is unrestricted by health problems [1]. In this modern society, where the average life expectancy is increasing, it is essential to extend not only life expectancy but also healthy life expectancy [2].

To extend healthy life expectancy, it is necessary to prevent frailty, a condition characterized by declines in physical and cognitive function. The main causes leading frailty are sarcopenia and poor quality of life (QoL) [3], both of which have been associated with reduced food intake. Gustatory function is known to decline with aging, and such age-related changes in taste perception have been reported to be associated with poor appetite and decreased energy intake in older adults [4]. This decline in taste sensitivity is attributed, in part, to reduced taste bud density and diminished function of taste receptor cells, along with other ageing-related factors [4]. However, findings on the association between taste sensitivity and frailty have been inconsistent. A study reported that self-reported taste impairment was associated with frailty, whereas objectively measured taste impairment was not [5], while another study in patients with chronic kidney disease reported an association between objectively measured taste impairment and frailty [6]. These discrepancies may be partly explained by differences in taste assessment methods and study populations.

Therefore, in the present study, we objectively assessed sweet, salt, and umami taste sensitivities in older Japanese adults and comprehensively examined their associations with frailty and health-related quality of life (HR-QoL). Furthermore, to elucidate the factors contributing to taste sensitivity, we examined its associations with diet quality and oral function in this population.

## 2 Methods

### 2.1 Study population and design

Participants were residents of a residential facility for older adults, PARK WELLSTATE Senri-Chuo, in Osaka, Japan, aged ≥60 years and independent in activities of daily living. This cohort study was initiated in 2023, and the current cross-sectional analysis used baseline data collected in 2023–2024 from participants who enrolled for the first time during this period. Participants were recruited through seminars and posters. No exclusion criteria were predefined for the entire cohort. However, one participant with taste impairment secondary to cerebral infarction was excluded from the current baseline analysis. Data on medical history and cognitive function, assessed using the revised version of the Hasegawa’s Dementia Scale (HDS-R), were obtained from health examination records.

### 2.2 Outcome assessments

Taste sensitivities for sweet, salt, and umami were evaluated using the whole mouth method. Sucrose solutions (FUJIFILM Wako Pure Chemical Corporation, Osaka, Japan) for sweet taste, sodium chloride solutions (FUJIFILM Wako Pure Chemical Corporation, Osaka, Japan) for salt taste, and monosodium L-glutamate solutions (NIPPON RIKA Corporation, LTD. Tokyo, Japan) for umami taste were prepared based on previous reports [7]. Cutoff values for low taste sensitivities were defined according to a previous study [7] (sweet ≥73 mmol/L; salt ≥210 mmol/L; umami ≥41 mmol/L).

Frailty was assessed using the Basic Checklist established by the Ministry of Health, Labour and Welfare (Supplemental Table S1) [8]. The total score ranges from 0 (no frailty) to 25 (severe frailty), with higher scores indicating poorer functioning. The score ≥8 indicates frailty.

HR-QoL was evaluated using the Medical Outcomes Study 36-Item Short Form Health Survey (SF-36), version 2 [9]. Each domain score ranges from 0 to 100, with higher scores indicating better HR-QoL.

Dietary intakes were examined using the self-administered long-food frequency questionnaire (FFQ) developed for Japanese individuals [10]. Diet quality was assessed using the Modified Japanese Diet Score (MJDS), which measures adherence to the traditional healthy Japanese dietary pattern, as described elsewhere [11] (Supplemental Table S2). MJDS ranges from 0 to 11, higher scores indicating better diet quality.

Oral function was evaluated by dentists according to established criteria, including assessments of tongue-lip motor function; oral uncleanness; oral dryness; decline in occlusal force; decline in tongue pressure; decline in chewing function; and decline in swallowing function [12]. Oral hypofunction was diagnosed when ≥three criteria were met.

### 2.3 Statistical analysis

Multiple regression analyses were conducted to examine the associations of taste sensitivity with frailty, HR-QoL, diet quality, and oral function. Analyses were adjusted for age (continuous), sex (male or female), and body mass index (BMI, continuous). The taste sensitivities were analyzed using each recognition threshold concentration. The normality of the data was evaluated using histograms and the Shapiro–Wilk test. Variables showing substantial deviation from normality, including taste sensitivities, Basic Checklist scores, SF-36 scores, and MJDS, were log-transformed. For SF-36, analyses were conducted among 64 participants with available data, and for MJDS, analyses were conducted among 59 participants with available data. Food intakes were adjusted for total energy intake. All statistical analyses were performed using R software (version 4.3.1, https://www.r-project.org/). A two-sided p value <0.05 was considered statistically significant.

## 3 Results

### 3.1 Clinical characteristics of participants

Forty participants in 2023 and additional 31 participants in 2024 agreed to participate in this study. One participant who developed a taste disorder after a stroke was excluded, resulting in 70 older adults included in the present study. The characteristics of the participants are shown in Supplemental Table S3. The mean ± standard deviation age was 82 ± 6 years, and mean BMI was 21.9 ± 3.2 kg/m^2^. The mean HDS-R score was 28.2 ± 2.2, and there were no participants with cognitive decline. Based on the Basic Checklist, 27.1% of participants were determined to have frailty. Oral hypofunction was identified in 60.4% of participants. The mean score of MJDS was 5.7 ± 1.8, which was slightly higher than that previously reported (4.1 ± 2.4) [11].

**Fig. 1:**
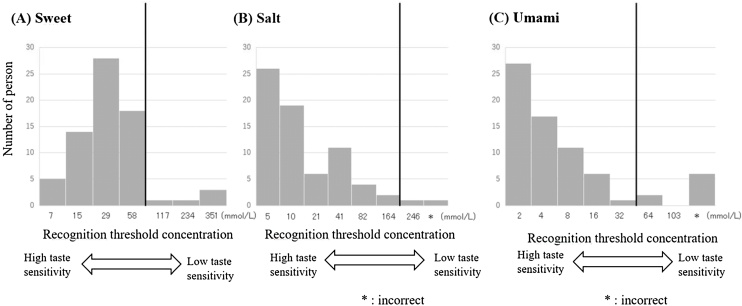
Histogram of recognition threshold concentration of three tastes.

The recognition threshold concentrations for three tastes (sweet, salt, and umami) are shown in Fig. 1. The median threshold concentrations were 29 mmol/L for sweet, 10 mmol/L for salt, and 4 mmol/L for umami, which were generally similar to previous reports (average threshold concentrations: 17 mmol/L for sweet, 21 mmol/L for salt, and 5.5 mmol/L for umami) [13]. Decreased taste sensitivity was observed in 7.1% of participants for sweet, 2.9% for salt, and 11.4% for umami.

### 3.2 Taste sensitivity and frailty

The associations between taste sensitivity and frailty, as assessed by the Basic Checklist, are shown in Table 1. After adjustment for age, sex, and BMI, lower sweet taste sensitivity was significantly associated with higher frailty scores (p = 0.004). No significant associations were observed between salt or umami taste sensitivity and Basic Checklist scores. Analyses of frailty subdomains indicated that lower sweet taste sensitivity was associated with lower functioning in the physical strength, memory, and mood domains (Supplemental Table S4).

**Table 1:** Taste sensitivity and Frailty / HR-QoL.

	Frailty		PCS		MCS	
N	70		64		64	
	Coefficient (95%CI)	P value	Coefficient (95%CI)	P value	Coefficient (95%CI)	P value
Sweet	0.28 (0.10, 0.46)	0.004	−0.04 (−0.16, 0.08)	0.51	−6.47 (−11.44, −1.50)	0.01
Salt	0.09 (−0.07, 0.24)	0.27	−0.09 (−0.19, 0.02)	0.10	−4.21 (−9.11, 0.69)	0.10
Umami	0.06 (−0.15, 0.27)	0.57	0.01 (−0.11, 0.13)	0.86	−5.89 (−11.40, −0.38)	0.04

Frailty was assessed by the Basic Checklist.

HR-QoL was assessed by SF-36.

The taste sensitivities were analyzed using each recognition threshold concentration.

Adjusted for age, sex, and BMI.

PCS: Physical component summary score, MCS: Mental component summary score.

### 3.3 Taste sensitivity and HR-QoL

The associations between taste sensitivity and HR-QoL are presented in Table 1. Lower sweet and umami taste sensitivities were significantly associated with lower mental component summary scores (p = 0.01 and 0.04, respectively). Analyses of SF-36 domains demonstrated that lower sweet taste sensitivity was associated with lower scores in the physical functioning, general health, social functioning, and mental health, and that lower umami taste sensitivity was associated with lower scores in the social functioning (Supplemental Table S5).

### 3.4 Modified Japanese diet score (MJDS) and taste sensitivity

To elucidate the factors related to better taste sensitivity, we examined the associations of taste sensitivity with diet and oral function. The associations between the MJDS and taste sensitivities of sweet, salt, and umami are shown in Table 2. After adjusting for age, sex, and BMI, the MJDS tended to be positively associated with sweet taste sensitivity (p = 0.09). No significant associations were observed for salt or umami taste sensitivity.

**Table 2:** Modified Japanese diet score (MJDS) / MJDS’s each food group and taste sensitivity.

	Sweet		Salt		Umami	
N = 59	Coefficient (95%CI)	P value	Coefficient (95%CI)	P value	Coefficient (95%CI)	P value
MJDS	−0.61 (−1.30, 0.07)	0.09	−0.46 (−1.24, 0.32)	0.26	−0.24 (−0.92, 0.45)	0.50
Whole grains	−0.15 (−0.34, 0.04)	0.12	−0.04 (−0.26, 0.17)	0.71	−0.11 (−0.29, 0.08)	0.27
Miso soup	0.06 (−0.14, 0.27)	0.54	−0.10 (−0.33, 0.13)	0.40	−0.28 (−0.47, −0.09)	0.005
Soybean products	−0.64 (−1.33, 0.06)	0.08	−0.70 (−1.48, 0.09)	0.09	0.16 (−0.54, 0.86)	0.66
Vegetables	−0.31 (−2.00, 1.39)	0.72	0.51 (−1.39, 2.41)	0.60	0.93 (−0.74, 2.59)	0.28
Mushrooms	−0.71 (−2.73, 1.30)	0.49	−0.19 (−2.46, 2.08)	0.87	1.61 (−0.37, 3.59)	0.12
Seaweeds	−0.42 (−1.77, 0.93)	0.54	−0.25 (−1.76, 1.27)	0.75	0.57 (−0.74, 1.89)	0.40
Fruits	−0.71 (−1.55, 0.12)	0.10	−1.19 (−2.10, −0.29)	0.01	−0.49 (−1.33, 0.34)	0.25
Fish and shellfish	1.33 (−0.34, 3.01)	0.13	−0.02 (−1.94, 1.90)	0.98	−0.01 (−1.68, 1.67)	0.99
Milk and dairy products	−0.21 (−1.07, 0.65)	0.64	−0.91 (−1.84, 0.03)	0.06	−0.41 (−1.25, 0.42)	0.34
High-sodium foods	0.01 (−0.29, 0.32)	0.93	−0.13 (−0.47, 0.21)	0.46	0.21 (−0.10, 0.51)	0.19
Green tea	−0.07 (−0.22, 0.07)	0.33	0.06 (−0.11, 0.22)	0.50	−0.03 (−0.18, 0.12)	0.70

The taste sensitivities were analyzed using each recognition threshold concentration.

High-sodium foods included pickled plums, pickled vegetables, dried fish, salted fish, and fish roe.

Adjusted for age, sex, and BMI.

In exploratory analyses examining each food group included in the MJDS (Table 2), miso soup consumption was positively associated with umami taste sensitivity (p = 0.005), and fruits consumption was positively associated with salt taste sensitivity (p = 0.01).

### 3.5 Oral function and taste sensitivity

Supplemental Table S6 presents the associations between oral hypofunction and taste sensitivity. Participants with oral hypofunction tended to have lower sweet taste sensitivity (p = 0.07). No significant associations were observed for salt or umami taste sensitivities.

## 4 Discussion

In the present study, we found that lower sweet taste sensitivity was significantly associated with frailty, and that lower sweet and umami taste sensitivities were significantly associated with lower mental HR-QoL. In addition, healthier dietary patterns and better oral function tended to be associated with better sweet taste sensitivity.

One possible mechanism underlying taste sensitivity and frailty is reduced appetite and subsequent sarcopenia due to decreased taste sensitivity [14,15]. Molecular mechanisms may also link taste receptor signaling to muscle maintenance. Sweet and umami tastes are mediated by the taste receptor type 1 (T1R) family, a family of G protein–coupled receptors consisting of T1R1, T1R2, and T1R3 [16]. Sweet taste is mediated by the T1R2/T1R3 receptor and umami taste is mediated by the T1R1/T1R3 receptor [16]. The T1R family is also expressed in organs other than the taste buds, including muscle [17]. Appropriate regulation of autophagy is essential for the maintenance of skeletal muscle mass and function, and reduced T1R3 signaling has been implicated in sarcopenia through autophagy dysregulation [16,18]. Moreover, myogenic regulatory factors (MRFs) such as MyoD regulate T1R3 expression in skeletal muscle, and inflammatory markers including NF-κB and TNF-α suppress MRF transcription and thereby inhibit myogenesis [19]. Since NF-κB and TNF-α increase with aging [20,21], age-related inflammation may downregulate T1R3 signaling and contribute to muscle degeneration. Collectively, these reports are consistent with the findings of the present study that reduced sweet taste sensitivity is associated with frailty.

Another possible contributor to frailty is low QoL [3]. Previous studies reported that subjective taste impairment was associated with lower QoL among older adults [22,23]. These reports are consistent with the findings of the present study, in which lower sweet and umami taste sensitivities were associated with lower mental QoL assessed by the SF-36. To our knowledge, this is the first study to objectively evaluate three taste sensitivities—sweet, salt, and umami—using the whole-mouth method and to investigate their association with HR-QoL assessed in older adults, whereas previous studies were subjective taste evaluations using self-administered questionnaires.

When we explored the factors contributing to better taste sensitivity, we found that higher Japanese diet quality and better oral function tended to be associated with better sweet taste sensitivity. Healthy dietary patterns—such as diets with high intakes of vegetables, fish, or healthy plant-based foods—have also been associated with better HR-QoL [24,25]. Furthermore, previous studies have shown that oral care program improved oral function in older adults [26,27]. Based on these past reports and the results of the present study, improving diet quality and oral function may have the potential to enhance taste sensitivity and reduce the risk of frailty.

Interestingly, in exploratory analyses, higher miso soup consumption was associated with better umami taste sensitivity. This may be related to habitual umami exposure, as miso soup contains monosodium glutamate, which has been reported to increase T1R3 expression in the tongue [28]. In addition, higher fruit consumption was associated with better salt taste sensitivity in this study. Although the underlying mechanism remains unclear, a previous study on the Mediterranean diet also reported a similar association [29]. Since diets rich in fruits and vegetables tend to be lower in processed foods, they may reduce habitual salt exposure and contribute to maintaining salt taste sensitivity [30]. These findings suggest that habitual dietary intake may be related to umami and salt taste sensitivity.

Several limitations should be noted. First, because of the cross-sectional design, it was not able to show the causal inferences. Longitudinal studies and interventional trials are warranted to confirm the directionality of the observed associations. Second, frailty, HR-QoL, and diet were assessed using self-administered questionnaires, which may produce measurement error. However, all questionnaires employed in this study have been validated and widely used in previous research [[8], [9], [10]]. Third, since all participants in this study were Japanese older adults residing in a single residential facility, the generalizability of our findings to other populations or settings may be limited.

## 5 Conclusion

In conclusion, the present study identified significant associations of sweet taste sensitivity with frailty and HR-QoL. Moreover, a healthier diet and better oral function tended to be associated with higher sweet taste sensitivity. These findings suggest that improving diet quality and oral function may enhance taste sensitivity and potentially contribute to the prevention of frailty in older adults.

## CRediT authorship contribution statement

Concept and design: SarK, MB. Project administration: MB. Investigation: SarK, MB, EY, SakK, TK, KM, AB, TH, SY, MashK, DK, MasaK, and MT. Data curation: SarK. Writing - original draft: SarK and MB. Writing - review & editing: EY, SakK, TK, KM, AB, NN, TH, CI, YH, MT, HN, JK, TT, SY, MashK, DK, MasaK, and IS. Supervision: MasaK and IS. MB had primary responsibility for final content. All authors read and approved the final manuscript.

## Ethics approval and consent to participate

The study was approved by the Institutional Ethics Review Board of Osaka University Hospital (approval number: 23008-3), and was conducted in accordance with the Declaration of Helsinki. Written informed consent was obtained from all participants.

## Declaration of Generative AI and AI-assisted technologies in the writing process

No AI tools were used in preparing this manuscript.

## Funding

None.

## Data availability

The data presented in this study are available on request from the corresponding author.

## Declaration of competing interest

All authors declare no conflict of interest.
