# Supplementary material for: Associations of taste sensitivity with frailty and health-related quality of life in older adults
Source: J Nutr Health Aging. 2026 Jan 24;30(3):100794. doi: 10.1016/j.jnha.2026.100794 (PMC12860929; doi:10.1016/j.jnha.2026.100794)
Supplement: Supplementary file 1 [file mmc1.docx]

**Supplemental Table 1.** The questions in the Basic Checklist

**Supplemental Table 2.** The questions in the Modified Japanese Diet Score

**Supplemental** **Table 3.** Clinical characteristics of participants

**Supplemental Table 4.** Associations between taste sensitivity and subcategories on the Basic Checklist

**Supplemental Table 5.** Associations between taste sensitivity and each domain score of HR-QoL

**Supplemental Table 6.** Associations between oral hypofunction and taste sensitivity

**Supplemental Table 1. The questions in the Basic Checklist**

| 1 | Do you go out by bus or train by yourself? | 0.Yes | 1.No |
| --- | --- | --- | --- |
| 2 | Do you go shopping to buy daily necessities by yourself? | 0.Yes | 1.No |
| 3 | Do you manage your own deposits and savings at the bank? | 0.Yes | 1.No |
| 4 | Do you sometimes visit your friends? | 0.Yes | 1.No |
| 5 | Do you turn to your family or friends for advice? | 0.Yes | 1.No |
| 6 | Do you normally climb stairs without using handrails or wall for support? | 0.Yes | 1.No |
| 7 | Do you normally stand up from a chair without any aids? | 0.Yes | 1.No |
| 8 | Do you normally walk continuously for 15 minutes? | 0.Yes | 1.No |
| 9 | Have you experienced a fall in the past year? | 1.Yes | 0.No |
| 10 | Do you have a fear of falling while walking? | 1.Yes | 0.No |
| 11 | Have you lost 2kg or more in the past 6 months? | 1.Yes | 0.No |
| 12 | Height: cm, weight: kg, BMI: kg/m^2^  If BMI is less than 18.5, this item is scored | 1.Yes | 0.No |
| 13 | Do you have any difficulties eating tough foods compared to 6 months ago? | 1.Yes | 0.No |
| 14 | Have you choked on your tea or soup recently? | 1.Yes | 0.No |
| 15 | Do you often experience having a dry mouth? | 1.Yes | 0.No |
| 16 | Do you go out at least once a week? | 0.Yes | 1.No |
| 17 | Do you go out less frequently compared to last year? | 1.Yes | 0.No |
| 18 | Do your family or your friends point out your memory loss? E.g. “You always ask the same question over and over again?” | 1.Yes | 0.No |
| 19 | Do you make a call by looking up phone numbers? | 0.Yes | 1.No |
| 20 | Do you find yourself not knowing today’s date? | 1.Yes | 0.No |
| 21 | In the last two weeks have you felt lack of fulfilment in your daily life? | 1.Yes | 0.No |
| 22 | In the last two weeks have you felt a lack of joy when doing the things you used to enjoy? | 1.Yes | 0.No |
| 23 | In the last two weeks have you felt difficulty in doing what you could do easily before? | 1.Yes | 0.No |
| 24 | In the last two weeks have you felt helpless? | 1.Yes | 0.No |
| 25 | In the last two weeks have you felt tired without a reason? | 1.Yes | 0.No |

1-5: Lifestyle, 6-10: Physical strength, 11-12: Nutrition, 13-15: Oral function, 16-17: Isolation, 18-20: Memory, 21-25: Mood

**Supplemental Table 2.** **The questions in the Modified Japanese Diet Score**

|  | Modified Japanese Diet Score |
| --- | --- |
| Food/Food Group | cut off |
| Whole grains | ≥3 times/week |
| Miso soup | ≥5 times/week |
| Soybean products | ≥3 times/week |
| Vegetables | ≥1.7 servings/day |
| Mushrooms | ≥3 times/week |
| Seaweeds | ≥3 times/week |
| Fruits | ≥3 times/week |
| Fish and shellfish | ≥3 times/week |
| Milk and dairy products | ≥5 times/week |
| High-sodium foods | <1 time/week |
| Green tea | ≥1 cup/day |
| Total score (points) | 11 |

**Supplemental Table 3. Clinical characteristics of participants**

|  | N=70 |
| --- | --- |
| Sex (male/female) | 20/50 |
| Age (years) | 82 ± 6 |
| Height (cm) | 155.5 ± 9.0 |
| Weight (kg) | 53.0 ± 9.9 |
| BMI (kg/m^2^) | 21.9 ± 3.2 |
| Frailty (the Basic Checklist) | 27.1% (19/70) |
| Oral hypofunction (n=48) | 60.4% (29/48) |
| Modified Japanese diet score (n=59) | 5.7 ± 1.8 |
| HDS-R (max 30 points) | 28.2 ± 2.2 |
| Diabetes (%, n=68) | 19.1% (13/68) |
| Hypertension (%, n=68) | 50.0% (34/68) |
| Dyslipidemia (%, n=68) | 44.1% (30/68) |
| PCS (n=64) | 41.1 ± 12.5 |
| MCS (n=64) | 55.6 ± 7.8 |

Data is presented as the mean value ± standard deviation or number or percentage of participants.

BMI: body mass index, HDS-R: version of Hasegawa’s Dementia Scale, PCS: physical component summary score, and MCS: mental component summary score.

**Supplemental Table 4. Associations between taste sensitivity and subcategories on the Basic Checklist**

| N=70 | Coefficient (95%CI) | P value |
| --- | --- | --- |
|  | Physical strength | |
| Sweet | 1.12 (0.35, 1.89) | 0.006 |
| Salt | 0.01 (-0.65, 0.67) | 0.97 |
| Umami | 0.46 (-0.38, 1.29) | 0.29 |
|  | Nutrition | |
| Sweet | 0.24 (-0.06, 0.55) | 0.12 |
| Salt | 0.03 (-0.22, 0.29) | 0.79 |
| Umami | 0.06 (-0.24, 0.36) | 0.71 |
|  | Oral function | |
| Sweet | 0.22 (-0.35, 0.78) | 0.45 |
| Salt | 0.11 (-0.33, 0.55) | 0.62 |
| Umami | 0.34 (-0.23, 0.92) | 0.25 |
|  | Isolation | |
| Sweet | 0.28 (-0.15, 0.70) | 0.21 |
| Salt | 0.37 (0.03, 0.71) | 0.04 |
| Umami | 0.08 (-0.36, 0.52) | 0.73 |
|  | Memory | |
| Sweet | 0.59 (0.11, 1.08) | 0.02 |
| Salt | 0.43 (0.04, 0.82) | 0.03 |
| Umami | -0.15 (-0.69, 0.38) | 0.58 |
|  | Mood | |
| Sweet | 1.24 (0.37, 2.10) | 0.007 |
| Salt | 0.57 (-0.17, 1.30) | 0.14 |
| Umami | 0.34 (-0.63, 1.31) | 0.49 |

Adjusted for age, sex, and BMI.

**Supplemental Table 5. Associations between taste sensitivity and each domain score of HR-QoL**

HR-QoL was assessed by SF-36. SF-36 consists of 36 questions measuring eight domains: physical function (PF), role physical (RP), bodily pain (BP), general health (GH), vitality (VT), social functioning (SF), role emotional (RE), and mental health (MH).

| *Physical QoL* | | | | | | | | |
| --- | --- | --- | --- | --- | --- | --- | --- | --- |
|  | PF | | RP | | BP | | GH | |
| N=64 | Coefficient (95%CI) | P value | Coefficient (95%CI) | P value | Coefficient (95%CI) | P value | Coefficient (95%CI) | P value |
| Sweet | -0.17 (-0.32, -0.02) | 0.03 | -0.06 (-0.16, 0.04) | 0.23 | -0.07 (-0.15, -0.0003) | 0.05 | -0.06 (-0.11, -0.009) | 0.02 |
| Salt | 0.02 (-0.12, 0.15) | 0.82 | -0.08 (-0.16, -0.003) | 0.046 | -0.01 (-0.08, 0.05) | 0.70 | -0.04 (-0.09, 0.002) | 0.06 |
| Umami | 0.005 (-0.17, 0.18) | 0.95 | -0.10 (-0.20, -0.001) | 0.05 | -0.07 (-0.15, 0.02) | 0.12 | -0.05 (-0.11, 0.004) | 0.08 |
| *Mental QoL* | | | | | | | | |
|  | VT | | SF | | RE | | MH | |
| N=64 | Coefficient (95%CI) | P value | Coefficient (95%CI) | P value | Coefficient (95%CI) | P value | Coefficient (95%CI) | P value |
| Sweet | -0.04 (-0.09, 0.02) | 0.17 | -0.12 (-0.19, -0.05) | 0.001 | -0.08 (-0.17, 0.01) | 0.09 | -0.07 (-0.11, -0.02) | 0.006 |
| Salt | -0.03 (-0.08, 0.01) | 0.18 | -0.02 (-0.08, 0.04) | 0.55 | -0.05 (-0.12, 0.03) | 0.22 | -0.02 (-0.06, 0.02) | 0.31 |
| Umami | -0.06 (-0.11, 0.003) | 0.07 | -0.11 (-0.18, -0.03) | 0.006 | -0.08 (-0.17, 0.008) | 0.08 | -0.02 (-0.08, 0.03) | 0.37 |

Adjusted for age, sex, and BMI.

**Supplemental Table 6. Associations between oral hypofunction and taste sensitivity**

|  | Sweet | | Salt | | Umami | |
| --- | --- | --- | --- | --- | --- | --- |
| N=48 | Coefficient (95%CI) | P value | Coefficient (95%CI) | P value | Coefficient (95%CI) | P value |
| Oral hypofunction | 0.37 (-0.02, 0.77) | 0.07 | -0.26 (-0.91, 0.39) | 0.44 | -0.07 (-0.55, 0.41) | 0.77 |

Multiple regression analyses were conducted to assess the associations between each taste sensitivity and oral function (presence or absence of oral hypofunction).

Adjusted for age, sex, and BMI.
